# ZKSCAN3 Upregulation and Its Poor Clinical Outcome in Uterine Cervical Cancer

**DOI:** 10.3390/ijms19102859

**Published:** 2018-09-20

**Authors:** Sun Lee, Young-Eun Cho, Joo-Young Kim, Jae-Hoon Park

**Affiliations:** 1 Department of Pathology, College of Medicine, Kyung Hee University, Seoul 130-701, Korea; leesun@khu.ac.kr (S.L.); pathology0277@naver.com (Y.-E.C.); 2 Radiation Oncology, National Cancer Center, 809 Madu-Dong, Ilsan-Koo, Goyang-si, Gyeonggi-Do 411-769, Korea; jooyoungcasa@ncc.re.kr

**Keywords:** ZKSCAN3, cervical cancer, oncoprotein

## Abstract

Zinc finger with KRAB and SCAN domain 3 (ZKSCAN3) upregulates genes encoding proteins involved in cell differentiation, proliferation and apoptosis. ZKSCAN3 has been reported to be overexpressed in several human cancers such as colorectal cancer and prostate cancer and is proposed as a candidate oncoprotein. However, the molecular mechanism by which ZKSCAN3 participates in carcinogenesis is largely unknown. Here, we evaluated ZKSCAN3 expression in uterine cervical cancers (CC) by immunohistochemistry using formalin-fixed, paraffin-embedded tissues from 126 biopsy samples from 126 patients. The clinicopathological findings were analyzed and compared with ZKSCAN3 expression levels. ZKSCAN3 was strongly overexpressed in CCs compared to adjacent non-neoplastic cervical mucosa tissues. Moreover, a gene copy number assay showed amplified ZKSCAN3 in CC samples. ZKSCAN3 overexpression was also significantly associated with poor overall survival of the patients. Overall, our findings indicate that ZKSCAN3 overexpression is a frequent event in uterine CC and is correlated with a poor clinical outcome. ZKSCAN3 could be developed as a molecular marker for prognostic prediction and early detection.

## 1. Introduction

Several decades ago, uterine cervical cancer (CC) was the leading cause of cancer-related deaths in women worldwide, but now ranks as the fourth-most common cause of cancer overall and the fourth-most common cause of death from cancer in women [1]. Nevertheless, the frequency of early cancers and precancerous lesions remains high, and CC is the second-most common cause of female-specific cancer after breast cancer [2]. Therefore, there has been extensive research focused toward the development of molecular markers for early cancer detection or predicting the patient’s prognosis.

Zinc finger with KRAB and SCAN domain 3 (ZKSCAN3) is a member of the KRAB and SCAN domain-containing zinc finger protein family, encoded by the *ZKSCAN3* gene in chromosome 6p22.1 [3]. This family of proteins is involved in cell differentiation, cell proliferation and apoptosis [3,4,5]. Recently, ZKSCAN3 was demonstrated to be a novel transcription factor that upregulates gene-coding proteins involved in cell growth, cell migration, angiogenesis and proteolysis [6]. For instance, ZKSCAN3 regulates integrin β4 expression, and integrin β4 knockdown reduced ZKSCAN3-augmented anchorage-independent colony formation [6]. Moreover, ZKSCAN3 has been reported as a novel driver of colorectal cancer progression [7] and to promote prostate cancer cell migration [8]. Collectively, these results suggest that ZKSCAN3 modulates the expression of genes favoring cancer progression. Although recent data suggest ZKSCAN3 as a robust oncoprotein, as well as transcriptional factor, the direct target of ZKSCAN3 remains to be identified, and its molecular mechanism during uterine cervical carcinogenesis is largely unknown.

Here, we analyzed ZKSCAN3 expression in uterine CC and adjacent non-malignant cervical mucosa. We further determined the relationship between ZKSCAN3 expression with patient clinicopathological characteristics. These results are expected to provide new insight into the mechanisms of cervical carcinogenesis and to highlight a potential new marker for the prognosis, diagnosis and/or treatment of CC.

## 2. Results

### 2.1. mRNA Expression of ZKSCAN3 in CC Cell Lines and Patient Samples 

**Figure 1:**
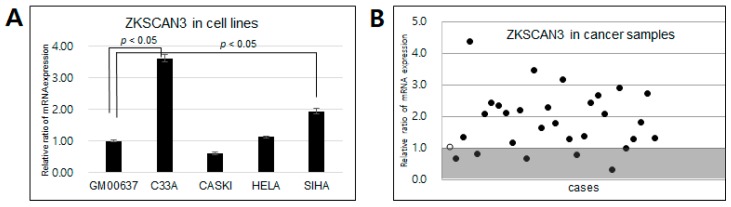
mRNA expression of *ZKSCAN3* in cervical cancer cell lines, human fibroblast (GM00637) and tissue samples from patients with cervical cancer. (**A**) qPCR targeting *ZKSCAN3* was performed with four cervical cancer cell lines (C33a, Caski, HeLa and SiHa). (**B**) Cervical cancer samples (closed circle) and normal fibroblasts (open circle) were analyzed by qPCR for *ZKSCAN3*.

We carried out quantitative polymerase chain reaction (qPCR) to determine the mRNA expression levels of *ZKSCAN3* in four cervical cancer cell lines (C33A, Caski, HeLa and SiHa), along with GM00637, a human fibroblast line, as a control sample. Moreover, we assessed *ZKSCAN3* expression in samples from 30 patients with CC. Figure 1A shows that *ZKSCAN3* expression was markedly upregulated in CC cell lines compared to human fibroblasts (GM00637), with significant upregulation detected for the C33A and SiHa cells (*p* < 0.05). Seventy seven percent (23/30 samples) of the CC patient samples also displayed upregulated *ZKSCAN3* expression compared to human fibroblasts (Figure 1B). Thus, the mRNA expression of *ZKSCAN3* is frequently upregulated in CC samples, as well as in CC cell lines.

### 2.2. ZKSCAN3 Protein Is Frequently Overexpressed in CC Tissues

**Figure 2:**
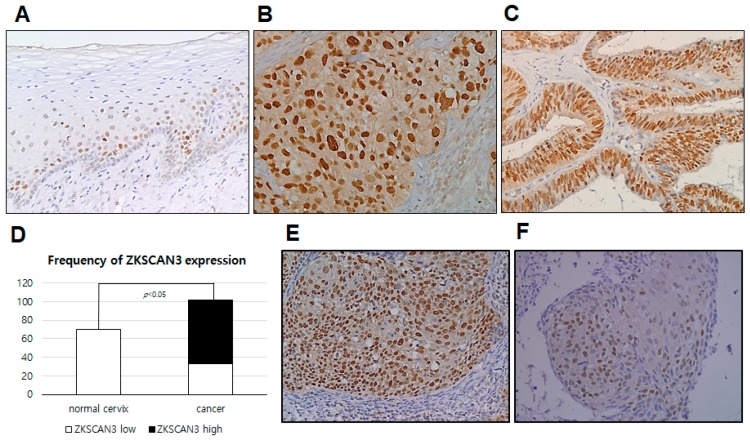
ZKSCAN3 expression in uterine cervical cancer and adjacent non-neoplastic mucosa. (**A**) ZKSCAN3 is rarely expressed in non-neoplastic squamous epithelial cells, except for basal cells. (**B**) ZKSCAN3 is strongly expressed at the nucleus of squamous cell carcinoma of the uterine cervix. (**C**) Cervical adenocarcinoma samples show strong expression of ZKSCAN3. (**D**) Frequency of ZKSCAN3 overexpression between cervical cancers and non-neoplastic cervical mucosae. (**E**) Strong nuclear expression of ZKSCAN3 in squamous cell carcinoma of the cervix. (**F**) Weak expression of ZKSCAN3 in squamous cell carcinoma of the uterine cervix. 200× magnification.

Next, we performed immunohistochemical staining with anti-ZKSCAN3 antibody to determine the protein expression using formalin-fixed paraffin-embedded tissues. Similar to the mRNA level results, ZKSCAN3 protein was found to be strongly expressed in the nucleus of tumor cells from all 126 CC samples (Figure 2B,C). Overall, 67 specimens had non-neoplastic cervical mucosa adjacent to cancer tissues for comparison, and 45 of these specimens (67%) showed weaker nuclear expression of ZKSCAN3 at the squamous epithelial cells, especially at the basal layer (Figure 2A). Compared to adjacent non-neoplastic cervical mucosa, the CC cells displayed a remarkably high frequency of ZKSCAN3 overexpression (Figure 2D). Next, to compare the expression grade, as well as expression frequency between cancer and non-neoplastic mucosa, we measured the scores of ZKSCAN3 expression based on both the intensity and proportion of expression, which were compared between CC tissues and non-neoplastic cervical mucosae. The CC samples demonstrated remarkably higher expression scores than non-neoplastic cervical mucosae (average 6.0 vs. 1.4, *p* < 0.05, Student *t*-test). Overall, 64% of the CC samples showed high-grade expression of ZKSCAN3 (scores > 5), whereas none of non-neoplastic cervical mucosa had scores this high. All 67 tissues with paired samples available showed exceedingly stronger expression of ZKSCAN3 in cancer than in the non-neoplastic cervical mucosa. These results demonstrate that ZKSCAN3 protein is frequently overexpressed in uterine CC compared to adjacent non-neoplastic cervical mucosa. 

### 2.3. Copy Number Variation of ZKSCAN3 in CC Patients 

**Figure 3:**
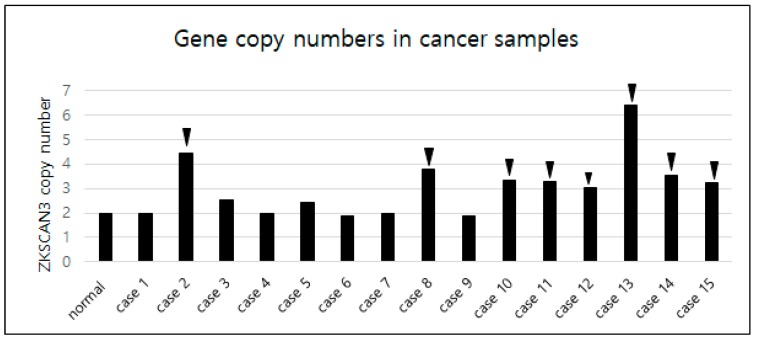
Gene copy number variation of *ZKSCAN3* determined using qPCR and normalized to the value of normal cervical samples. Arrow heads indicate the cases with increased copies of *ZKSCAN3*.

Given the clear overexpression of ZKSCAN3 in CC, we next explored the potential causative mechanism. Gene copy number variation of *ZKSCAN3* was analyzed using qPCR from genomic DNA of the 15 formalin-fixed and paraffin-embedded CC patient samples and 10 normal cervical tissue samples. Normal cervical tissues diagnosed as chronic cervicitis were included as the calibrator sample (calibrator value = 2). The cycle threshold (Ct) value variation of the housekeeping gene was low, and the coefficient of variation was less than 5%. Interestingly, 53% (8/15) of the samples exhibited increased copies of *ZKSCAN3*, whereas 47% of the samples showed the expected two copies; no gene deletion was detected in any sample (Figure 3). All eight samples with increased copies of *ZKSCAN3* showed high-grade expression of ZKSCAN3 (scores > 5), whereas one out of six samples without amplification displayed high-grade ZKSCAN3 expression. This result suggests that amplification of *ZKSCAN3* is one of the causative mechanisms of ZKSCAN3 overexpression in CC.

### 2.4. ZKSCAN3 Promotes Cell Invasion in CC Cells

**Figure 4:**
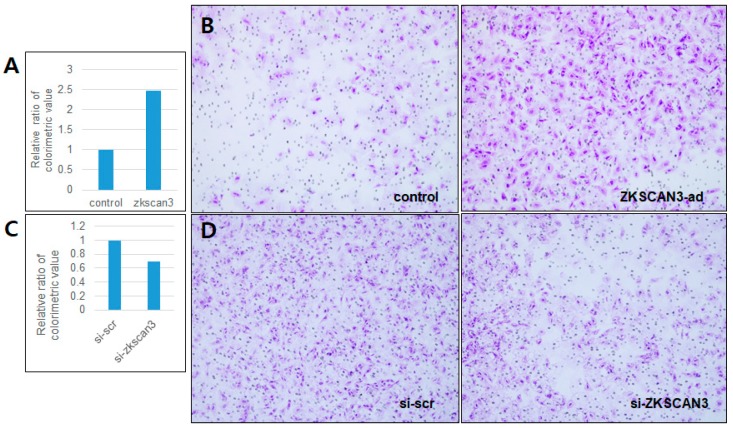
ZKSCAN3 expression affects the invasion ability of HeLa cells. (**A**,**C**) HeLa cells were transduced with ZKSCAN3-HA adenovirus for 24 h and with siRNA for 48 h, respectively, and then, the transwell invasion assay was performed. (**B**,**D**) Representative fields of view of each group of cells (hematoxylin stain, 100× magnification).

To clarify the impact of ZKSCAN3 overexpression on CC cells, we carried out a transwell invasion assay using the HeLa cell line, which was chosen because it showed a relatively basal level of ZKSCAN3 expression compared to the other cell lines (Figure 1A), allowing for the effect of ZKSCAN3 overexpression to be clearly determined. As expected, ZKSCAN3 overexpression promoted the invasion ability (Figure 4A,B), whereas ZKSCAN3 knockdown reduced the invasion ability of HeLa cells (Figure 4C,D). These results suggest that ZKSCAN3 plays an important role in promoting the tumor aggressiveness of CCs and likely functions as a robust oncoprotein. 

### 2.5. Correlation of ZKSCAN3 Expression and Clinicopathological Findings of the Patients with Cervical Cancers

The clinicopathological characteristics of the 126 patients with CC were compared with the immunohistochemical results along with follow-up data, including the status of survival and disease recurrence, overall survival time and disease-free survival time. The mean follow-up period was 44.1 months (6–124 months).

As shown in Table 1, 117 (91%) of the patients were positive for human papillomavirus, and type 16 was the most frequent genotype (36%). To determine the relationship between ZKSCAN3 expression and the clinicopathological features, the CC cases were divided into two groups: those with low expression tumors (score of five or less) and those with high expression tumors (score of six or more) (Figure 2E,F). As shown in Table 2, the overall survival period and disease-free period were significantly shorter in cancers with high-grade ZKSCAN3 than in those with low grade tumors (*p* = 0.001 and 0.002, respectively, Student *t*-test). Other clinicopathological parameters, including age, FIGO stage, presence or absence of pelvic lymph node metastasis, tumor recurrence or distant metastasis, were not associated with the expression grade of ZKSCAN3. ZKSCAN3 overexpression remained an independent prognostic factor along with FIGO stage in the multivariate analysis (Table 3). We also analyzed the correlation to HPV genotypes or viral titers, but significant findings were not observed. 

**Figure 5:**
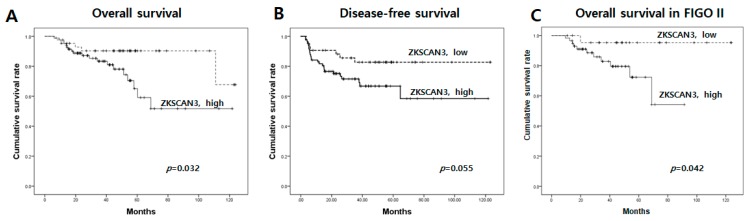
Overall survival and disease-free survival curves analyzed by the Kaplan–Meir log rank test, showing that high-grade of ZKSCAN3 overexpression is associated with a shorter survival time in patients with uterine cervical cancers. (**A**) Comparison of overall survival rate of patients with low- or high-grade ZKSCAN3 expression. (**B**) Comparison of disease-free survival rate of patients with low- or high-grade ZKSCAN3 expression. (**C**) Overall survival rate of the patients in FIGO stage II with low- or high-grade ZKSCAN3 expression.

Next, we analyzed the cumulative survival rates using the Kaplan–Meier log-rank test and compared the results from the low- and high-grade ZKSCAN3 expression groups. Interestingly, overall survival, as well as disease-free survival were superior in the patients with low-grade ZKSCAN3 expression, although the latter difference was just short of reaching statistical significance (Figure 5A,B). Moreover, when the analysis was confined to the cases at FIGO stage II, the overall survival was remarkably poorer in the patients with high-grade ZKSCAN3 expression (Figure 5C). These results demonstrate that ZKSCAN3 overexpression is strongly associated with the poor prognosis of uterine CCs.

## 3. Discussion

ZKSCAN3 is a candidate oncoprotein, and its expression is frequently upregulated in human cancers such as colorectal cancer, prostate cancer, bladder cancer and multiple myeloma [3,7,8,9,10,11] Yang et al. [7] demonstrated that *ZKSCAN3* was overexpressed in colorectal tumor tissue compared with adjacent nonmalignant mucosa, determined by RT-PCR, which was found to be partly due to gene amplification based on Southern blotting analysis. Moreover, ZKSCAN3 knockdown in colon cancer cell lines resulted in impaired anchorage-independent growth and orthotopic tumor growth, whereas overexpression of ZKSCAN3 showed the opposite effect and increased 5-fluorouracil resistance [7].

Recently, ZKSCAN3 was identified as a novel transcription factor that upregulates gene-coding proteins involved in growth (MEK2, guanine nucleotide exchanger RasGRP2, insulin-like growth factor-2 and integrin β4), cell migration (MST1R), angiogenesis (vascular endothelial growth factor) and proteolysis (MMP26, cathepsin D and PRSS3) [6]. Specifically, ZKSCAN3 overexpression or silencing modulated integrin β4 expression and integrin β4 knockdown by small hairpin RNA reduced the ZKSCAN3-augmented anchorage independent colony formation. The authors suggested that ZKSCAN3 regulates the expression of the genes favoring tumor progression. Despite these recent data strongly suggesting that ZKSCAN3 is a robust oncoprotein and transcriptional factor, the direct target of ZKSCAN3 remains to be identified, and its molecular mechanism during carcinogenesis is largely unknown. 

Here, we demonstrated that ZKSCAN3 was remarkably overexpressed in 126 tissue samples of uterine CCs compared to non-neoplastic cervical mucosa. Moreover, cervical intraepithelial lesions included in biopsy samples showed frequent ZKSCAN3 overexpression. Thus, ZKSCAN3 likely plays an important role in the multistep carcinogenesis of CC, and its overexpression is an early event during the process.

Detection of gene copy number variation of *ZKSCAN3* using qPCR is the most feasible assay to detect gene amplification. We analyzed gene copy number variation of ZKSCAN3 from gDNA extracted from formalin-fixed paraffin-embedded tissues of patients’ samples and demonstrated a frequent event of *ZKSCAN3* amplification in CCs. This method can thus be developed as a screening test of *ZKSCAN3* amplification in CCs, which will require further evaluation at a larger scale to verify the clinical significance of this pattern. 

Analysis of the association between ZKSCAN3 overexpression and the clinical outcome of CC patients showed that ZKSCAN3 overexpression, as well as FIGO stage are poor prognostic factors. Moreover, our in vitro study demonstrated that ZKSCAN3 overexpression promoted the invasion ability of cancer cells. These results strongly suggest that ZKSCAN3 plays a role in tumor progression, as well as in tumor development. Therefore, ZKSCAN3 could be developed as an emerging marker for the early detection of uterine CC in the field of cytology or biopsy and for predicting a poor clinical outcome.

In conclusion, ZKSCAN3 overexpression is frequently observed in uterine CCs and is associated with a poor clinical outcome. Thus, our results suggest that ZKSCAN3 plays an important role in the multistep carcinogenesis and progression of uterine CC.

## 4. Materials and Methods

### 4.1. Samples and Clinical Characteristics

For the immunohistochemical analysis of ZKSCAN3, formalin-fixed and paraffin-embedded tissues (126 CC samples) were obtained from the Center for Uterine Cancer, National Cancer Center, Gyeonggi-Do, South Korea. Tumor staging and clinicopathological classification were performed according to the international federation of Obstetrics and Gynecology, 2009 (FIGO), classification of carcinoma of the uterine cervix. For the evaluation of mRNA expression of *ZKSCAN3*, 30 frozen tissues of CC were available among 126 CC samples. For the analysis of gene copy number variation of *ZKSCAN3*, 15 CC samples were available to obtain a sufficient amount of genomic DNA from formalin-fixed paraffin-embedded tissues among 126 CC samples. In addition, 10 normal cervical tissues diagnosed as chronic cervicitis were used as a normal control. All patients provided informed consent for analysis of their tissues for research purposes, and the study was approved by Ethics Committee and Institutional Review Board of National Cancer Center (No. NCC 20160019, 29 January 2016).

### 4.2. Immunohistochemical Analysis

Immunohistochemical staining was performed to determine the expression level of ZKSCAN3 in CC samples as previously described [12]. In brief, antigen retrieval of the tissues after deparaffinization was carried out using 10 mM sodium citrate buffer (pH 6.0). The slides were treated with 0.45% hydrogen peroxide for 20 min to block the endogenous peroxidase activity and then incubated with 2.5% normal horse serum at room temperature for 20 min. Next, the tissues were incubated with primary antibody against ZKSCAN3 (HPA00637, Sigma, 1:200) overnight at 4 °C. After washing with phosphate-buffered saline, the slides were incubated using the Dako REAL^TM^ EnVision^TM^ detection system (DAKO, Glostrup, Denmark) according to the manufacturer’s protocol. The slides were then treated with Dako REAL^TM^ DAB+ chromogen (DAKO, Glostrup, Denmark) for 2 min and counterstained with 5% Mayer’s hematoxylin solution. The tissues were dehydrated and mounted. 

Evaluation of the expression pattern of ZKSCAN3 was conducted independently by two pathologists (Park J.H. and Lee S.). The degree of protein expression was scored according to the proportion of positively-stained tumor cells and the intensity of the staining. The proportion and intensity scores were multiplied (0–12), and the resultant scores were divided into two groups: low for scores below 6 and high for scores greater than 6. 

### 4.3. Cell Culture, Plasmid Construction and Transfection

As previously described, cells were cultured in Dulbecco’s Modified Eagle’s Medium supplemented with 10% fetal bovine serum (FBS) and 1% antibiotics [13]. ZKSCAN3-HA adenovirus was purchased from ABM Inc. (San Jose, CA, USA). Human ZKSCAN3 siRNA was purchased from Santa-Cruz Biotech (Santa Cruz, CA, USA, sc-95093). Cells were transfected with siRNA using Oligofectamine^TM^ (Invitrogen, Carlsbad, CA, USA) according to the manufacturer’s recommendations. 

### 4.4. Quantitative Real-Time PCR for mRNA Expression

CC cell lines (HeLa, C33a, Caski and SiHa), human fibroblast (GM00637) and 30 CC frozen samples were analyzed by qPCR for *ZKSCAN3* and *β-actin* (*ACTB*). For comparison, the values of CC cell lines or CC clinical samples were calibrated to the value of human fibroblasts (GM00637). Only 30 frozen samples among 126 CC samples were available for RNA extraction. We converted 1 µg of total RNA to cDNA as previously described [13]. The sequences of the gene-specific primers are as follows: *ZKSCAN3* forward primer 5′-GGTCTCCCTGGGTGATGAAA-3′, reverse primer 5′-GCACATGTAGGAATCTGGGC-3′, *ACTB* forward primer 5′-TGAACCCCAAGGCCAACCGC-3′, reverse primer 5′-GACCCCGTCACCGGAGTCCA-3′. PCR was performed using the StepOnePlus real-time PCR system (Applied Biosystems^TM^, Applied Foster City, CA, USA) according to the manufacturer’s manual. 

### 4.5. Quantitative PCR for Copy Number Aberration Detection

Genomic DNA was extracted from the formalin-fixed paraffin-embedded tissues of 15 patients among 126 CC patients and 10 normal cervical tissues. For each PCR, a total of 5 ng of genomic DNA from patient samples was used. TaqMan genotyping master mix was used with primer sets targeting *ZKSCAN3* (HS00197508) and reference genes according to the manufacturer’s protocol (Thermo Fisher Scientific, Rockford, IL, USA). The reaction mixture was prepared, and amplification was performed in a system under the following condition: hold 10 min at 95 °C and then 40 cycles of 15 s at 95 °C and 60 s at 60 °C. *GAPDH* and *ACTIN* as housekeeping genes were used for the quantification of the *ZKSCAN3* copy number variation (CNV) status in this study. Data were analyzed by the CopyCaller software V2.1 (Thermo Fisher Scientific, Rockford, IL, USA). The CNV of *ZKSCAN3* was calculated using the Ct value with the following formula: CNV = 2^−ΔΔ*C*t^, where ΔΔ*C*t = (Ct_,target_ − Ct_,reference_)_CC sample_ − (Ct_,target_ − Ct_,reference_)_normal cervical sample_ [14]. The target represents *ZKSCAN3*, and the reference represents the housekeeping gene. To define the normal *ZKSCAN3* gene copy number relative to the two reference genes in our test, we used 10 normal cervical mucosae as a normal control. A gene copy number ratio of the unknown sample ≥3 was defined as amplified. A gene copy number ratio of the unknown sample <3 was defined as non-amplified [15].

### 4.6. Invasion Assay

As previously described, the invasion assay was performed using a cell invasion kit (Chemicon, Billerica, MA, USA) according to the manufacturer’s protocol. In brief, 1 × 10^4^ cells were plated on Matrigel-coated membranes in transwell invasion chambers and incubated at 37 °C for 24 h. Invading cells were fixed with methanol and stained with hematoxylin. Three independent invasion assays were performed in triplicate.

### 4.7. Statistical Analysis

Statistical analyses were carried out using SPSS software, Version 13.0 (IBM, Chicago, IL, USA). Data were analyzed with Pearson’s chi-squared test. Differences with *p*-values of less than 0.05 were considered statistically significant.

## Figures and Tables

**Table 1:** Clinicopathological characteristics of the 126 patients with cervical cancers.

Clinicopathological Findings	Number of Cases (%)
Age (average)	26–79 years (53.2)
FIGO stage	
I	24 (19.0)
II	80 (63.5)
III	17 (13.5)
IV	5 (4.0)
Pelvic lymph node metastasis	
No	47 (37.3)
Yes	79 (62.7)
Survival	
Alive	102 (81.0)
Death	24 (19.0)
Tumor recurrence or distant metastasis	
No	94 (74.6)
Yes	32 (25.4)

**Table 2:** Expression of ZKSCAN3 in cervical cancer: correlation with the clinicopathological findings.

	ZKSCAN3		*p*-Value *
Clinicopathological Findings	Low Grade (%)	High Grade (%)	
Age (average)	53.4 years	53.1 years	0.44
FIGO stage			0.40
I	10 (23.3)	14 (16.9)	
II	23 (53.5)	56 (68.7)	
III	8 (18.6)	9 (10.8)	
IV	2 (4.7)	3 (3.6)	
Pelvic lymph node metastasis			0.43
No	14 (32.6)	33 (39.8)	
Yes	29 (67.4)	50 (60.2)	
Survival			0.16
Alive	38 (88.4)	64 (77.1)	
Death	5 (11.6)	19 (22.9)	
Tumor recurrence or distant metastasis			0.13
No	36 (83.7)	58 (69.9)	
Yes	7 (16.3)	25 (30.1)	
Overall survival (average, months)	55.2 months	38.4 months	0.001
Disease free survival (average, months)	47.9 months	32.3 months	0.002
5-year survival rate	90.3%	59.2%	0.03

* Analyzed by Student *t*-test, Pearson chi-square test or Fisher’s exact test.

**Table 3:** Multivariate analysis of clinicopathological prognostic factors in the patients with uterine cervical cancer.

Clinicopathological Factors	Overall Survival	
	Relative Risk (95% Confidence Interval)	*p*-Value *
Age	0.40–2.14	0.85
Pelvic LN metastasis	0.17–1.24	0.45
FIGO stage	1.20–3.09	0.01
ZKSCAN3 overexpression	1.10–8.37	0.03

* Multivariate analysis by the Cox multiple regression model after backward stepwise elimination with variables eliminated at *p* < 0.1.

## References

[1] World Health Organization (2014). World Cancer Report 2014.

[2] World Health Organization (2014). World Cancer Report 2014.

[3] Wang H., Yang L., Jamaluddin M.S., Boyd D.D. (2004). The Kruppel-like KLF4 transcription factor, a novel regulator of urokinase receptor expression, drives synthesis of this binding site in colonic crypt luminal surface epithelial cells. J. Biol. Chem..

[4] Urrutia R. (2003). KRAB-containing zinc-finger repressor proteins. Genome Biol..

[5] Lupo A., Cesaro E., Montano G., Zurlo D., Izzo P., Costanzo P. (2013). KRAB-zinc finger proteins: A repressor family displaying multiple biological functions. Curr. Genom..

[6] Yang L., Zhang L., Wu Q., Boyd D.D. (2008). Unbiased screening for transcriptional targets of ZKSCAN3 identifies integrin beta-4 and vascular endothelial growth factor as downstream targets. J. Biol. Chem..

[7] Yang L., Hamilton S.R., Sood A., Kuwai T., Ellis L., Sanguino A., Lopez-Berestein G., Boyd D.D. (2008). The previously undescribed ZKSCAN3 (ZNF306) is a novel ‘driver’ of colorectal cancer progression. Cancer Res..

[8] Zhang X., Jing Y., Qin Y., Hunsucker S., Meng H., Sui J., Jiang Y., Gao L., An G., Yang N. (2012). The zinc finger transcription factor ZKSCAN3 promotes prostate cancer cell migration. Int. J. Biochem. Cell Biol..

[9] Abdul Murad N.A., Razak Z.A., Hussain R.M., Syed Hussain S.N., Ching Huat C.K., Che Md Ali S.A., Norlia A., Rohaizak M., Naqiyah I., Rahman J. (2013). Quantification of Her-2/Neu gene in breast cancer patients using real time-polymerase chain reaction (Q-PCR) and correlation with immunohistochemistry findings. Asian Pac. J. Cancer Prev..

[10] Kim C.W., Roh S.A., Tak K.H., Koh B.M., Ha Y.J., Cho D.H., Kim S.-Y., Kim Y.S., Kim J.C. (2016). ZKSCAN3 facilitates liver metastasis of colorectal cancer associated with CEA-expression tumor. Anticancer Res..

[11] Kawahara T., Inoue S., Ide H., Kashiwagi E., Ohtake S., Mizushima T., Li P., Li Y., Zheng Y., Uemura H. (2016). ZKSCAN3 promotes bladder cancer cell proliferation, migration, and invasion. Oncotarget.

[12] Yang L., Wang H., Kornblau S.M., Graber D.A., Zhang N., Matthews J.A., Wang M., Weber D.M., Tomas S.K., Shah J.J. (2011). Evidence of a role for the novel zinc-finger transcription factor *ZKSCAN3* in modulating *Cyclin D2* expression in multiple myeloma. Oncogene.

[13] Lee S., Cho Y.E., Kim Y.J., Park J.H., Jun C. (2016). N-terminal kinase regulates the nucleoplasmic translocation and stability of nucleolar GLTSCR2 protein. Biochem. Biophys. Res. Commun..

[14] Lee S., Kim J.Y., Kim Y.J., Seok K.O., Kim J.H., Chang Y.J., Kang H.Y., Park J.H. (2012). Nucleolar protein GLTSCR2 stabilizes p53 in response to ribosomal stresses. Cell Death Differ..

[15] Livak K.J., Schmittgen T.D. (2001). Analysis of relative gene expression data using real-time quantitative PCR and the 2^−ΔΔ*C*t^ Method. Methods.

